# Computational Dissection of the Role of Trp305 in the Regulation of the Death-Associated Protein Kinase–Calmodulin Interaction

**DOI:** 10.3390/biom12101395

**Published:** 2022-09-29

**Authors:** Yu-Ping Zhu, Xin-Yi Gao, Guo-Hui Xu, Zhao-Fu Qin, Hai-Xing Ju, De-Chuan Li, De-Ning Ma

**Affiliations:** 1 Department of Colorectal Surgery, The Cancer Hospital of the University of Chinese Academy of Sciences (Zhejiang Cancer Hospital), 1 Banshan East Road, Hangzhou 310022, China; 2 Institute of Basic Medicine and Cancer (IBMC), Chinese Academy of Sciences, 1 Banshan East Road, Hangzhou 310022, China; 3 Department of Radiology, The Cancer Hospital of the University of Chinese Academy of Sciences (Zhejiang Cancer Hospital), 1 Banshan East Road, Hangzhou 310022, China

**Keywords:** death-associated protein kinase 1 (DAPK1), calmodulin, molecular dynamics simulations, principal component analysis, binging free energy calculation

## Abstract

Death-associated protein kinase 1 (DAPK1), as a calcium/calmodulin (CaM) regulated serine/threonine kinase, functions in apoptotic and autophagy pathways and represents an interesting drug target for inflammatory bowel disease and Alzheimer’s disease. The crystal structure of the DAPK1 catalytic domain and the autoregulatory domain (ARD) in complex with CaM provides an understanding of CaM-dependent regulation of DAPK1 activity. However, the molecular basis of how distinct Trp305 (W305Y and W305D) mutations in the ARD modulate different DAPK1 activities remains unknown. Here, we performed multiple, μs-length molecular dynamics (MD) simulations of the DAPK1–CaM complex in three different (wild-type, W305Y, and W305D) states. MD simulations showed that the overall structural complex did not change significantly in the wild-type and W305Y systems, but underwent obvious conformational alteration in the W305D system. Dynamical cross-correlation and principal component analyses revealed that the W305D mutation enhanced the anti-correlated motions between the DAPK1 and CaM and sampled a broader distribution of conformational space relative to the wild-type and W305Y systems. Structural and energetical analyses further exhibited that CaM binding was unfavored in response to the W305D mutation, resulting in the decreased binding of CaM to the W305D mutant. Furthermore, the hydrogen bonds and salt bridges responsible for the loss of CaM binding on the interface of the DAPK1–CaM complex were identified in the W305D mutant. This result may provide insights into the key role of Trp305 in the regulation of CaM-mediated DAPK1 activity.

## 1. Introduction

As the superfamily of calcium/calmodulin (CaM) regulated serine/threonine kinases (CaMKs), the death-associated protein kinase (DAPK) family regulates important biological functions in human cells, including autophagy, apoptosis, membrane blebbing, tumor-necrosis factor-mediated cell death, disassociation from extracellular matrix erythropoiesis, granulocyte maturation, and tumor metastasis [1,2,3]. DAPK is the prototype of a five-member family of CaMKs that contains DAPK1–DAPK3 and DAPK-related apoptosis-inducing protein kinases 1 and 2 (DRAK1 and DRAK2, also called STK17A and STK17B, respectively) [3]. Among them, DAPK1 has 1430 residues and is the largest member of the DAPK family based on the size. It is well known for its role as an efficient tumor suppressor and a regulator of apoptosis and autophagy [3,4,5,6], and is an interesting drug target for Alzheimer’s disease and inflammatory bowel disease [7,8]. Thus, DAPK1 has been the subject of an increasing number of investigations compared to other members.

Decades of intensive research and the accumulation of structural and functional data elucidates that DAPK1 is a large multi-domain protein that includes an N-terminal kinase domain, a CaM-binding domain, an ankyrin repeats domain, a cytoskeleton binding domain, and a carboxyl-terminal death domain [3]. In physiological conditions, DAPK1 is auto-phosphorylated at Ser308 located at the CaM-binding domain [9,10]. The resulting auto-phosphorylation of Ser308 (pSer308) prevents the binding of CaM to the phosphorylated DAPK, which renders it in the inactive state. Upon stimulation, the class III phosphoinostitide 3-kinase-dependent phosphatase (PI3K) dephosphorylatespSer308 of DAPK1 and promotes CaM binding to the CaM-binding domain. This outcome relieves the auto-inhibition of DAPK1 and keeps it in the active state, which triggers apoptosis responses.

**Figure 1:**
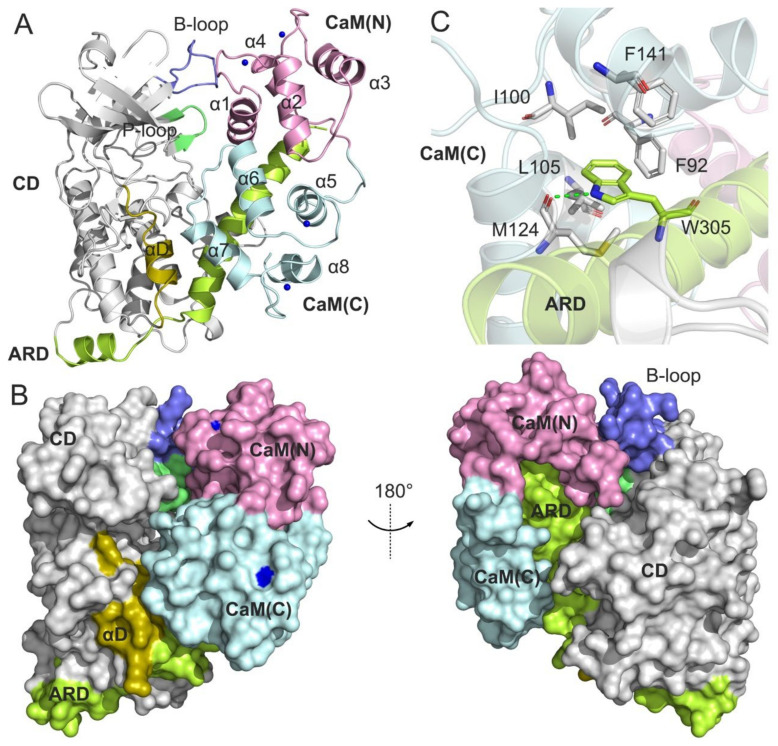
(**A**) A representation of the structural complex of DAPK1–CaM (PDB ID: 2X0G). The catalytic domain (CD) and the autoregulatory domain (ARD) of DAPK1 are colored gray and lemon, respectively. The important basic loop (B-loop), the phosphate-binding loop (P-loop), and the helix αD are colored light blue, lime, and olive, respectively. The N-terminal and the C-terminal CaM are colored pink and light cyan, respectively. Calcium ions are shown by blue spheres. (**B**) Surface representation of the structural complex of DAPK1–CaM. (**C**) The hydrophobic network of Trp305 with the CaM residues. Hydrogen bonds are shown by green dotted lines.

Although a large number of DAPK1 crystal structures have been reported to date, the full-length structures are still unavailable. The determination of an X-ray crystal structure of the DAPK1 catalytic domain and the autoregulatory domain in complex with CaM (PDB ID: 2X0G) unequivocally ascertains the association of the unphosphorylated DAPK1 with CaM (Figure 1), which provides an understanding of the molecular mechanism for the role of CaM in the regulation of DAPK1 catalytic activity [11]. Biochemical assays have showed that mutation of Trp305 at the ARD to a tyrosine (W305Y) only moderately decreased CaM binding, whereas mutation of Trp305 to an aspartate (W305D) significantly reduced CaM binding [11]. Moreover, the DAPK1 W305D mutant exhibited an obvious increase in Ser308 autophosphorylation, suggesting the critical role of Trp305 in the regulation of DAPK1 activity. However, the molecular basis of the effect of the distinct Trp305 variations on the DAPK1–CaM interaction is still unknown.

Molecular dynamics (MD) simulations based on the static crystal structures can provide valuable information regarding the ligand–protein or protein–protein interactions and capture the dynamic conformational changes of biomacromolecules induced by mutations [12,13,14,15,16]. Thus, MD simulations have now become an important tool to connect the static snapshot of the crystallography with the kinetic information, promoting the detailed understanding of protein conformational transitions at the atomic level [17,18,19,20,21]. Here, we performed multiple μs-length MD simulations of the DAPK1–CaM complex in three different (wild-type, W305Y, and W305D) states. Structural and energetical analyses were then used to decipher the effect of Trp305 variations on the conformational dynamics of the DAPK1–CaM complex. This result is expected to advance our insights into the key role of Trp305 in modulating DAPK1 activity.

## 2. Materials and Methods

### 2.1. Construction of Simulation Systems

The X-ray crystal structure of the DAPK1 catalytic domain and the autoregulatory domain (aa 3–320) in complex with CaM (aa 6–145) (PDB ID: 2X0G) [11] was downloaded from the RCSB Protein Data Bank. The missing residues 74–81 in the CaM crystal structure were modelled using CHARMM-GUI [22]. For the W305Y and W305D mutants, corresponding residues were mutated.

### 2.2. MD Simulations

MD simulations in explicit water were performed using the AMBER 18 package [23]. The Amber ff14SB force field was used for the protein [24] and the TIP3P model was used for water molecules [25]. For each system, the initial structure was solvated in an octahedral box solvated with ~18,765 TIP3P water molecules with an edge distance of 10.0 Å. There were 58–59 Na^+^ and 44 Cl^−^ counter ions added to ensure neutrality at an ionic strength of 0.15 M, resulting in a box of around 109 × 109 × 109 Å^3^ using periodic boundary conditions.

First, each system was related through energy minimization to ensure that the system was free from steric clashes. The minimization was performed using a combination of the steepest descent and conjugate gradient methods. Then, each system was heated to 300 K in 500 ps with a positional restraint of 10 kcal mol^−1^ Å^−2^ for all solute atoms. This was followed by equilibration simulations at 300 K in 1 ns with a positional restraint of 10 kcal mol^−1^ Å^−2^ for all solute atoms under the canonical ensemble (NVT). A 2 fs time step was used for the equilibration and production simulations. In the production stage, three independent replicates of 1 μs MD simulations for each system was performed with random velocities under the isothermal isobaric (NPT) ensemble. The particle mesh Ewald (PME) method was used to evaluate long-range electrostatic interactions with grid-spacing of 10 Å [26] and the SHAKE constraint algorithm was used to constrain all covalent bonds involving hydrogen atoms [27]. The temperature (300 K) and pressure (1 bar) were coupled with a time constant of 1.0 ps using the Langevin algorithm [28].

### 2.3. Principal Component Analysis (PCA)

The principal component analysis (PCA) was performed on the Cartesian coordinates of protein Cα atoms evolved along the simulation time course [29,30]. The protein backbone atoms from the simulated trajectories were superimposed to that of the reference structure (zeroth frame). The first few principal component (PCs) were regarded to interpret the essential dynamics [31]. The first principal component (PC1) represents the major conformational dynamics, followed by other components. To uncover the dynamical states, hierarchical clustering was done in PC space. This yields prominent subgroups. The subgroups’ average structures reflect different conformational states.

### 2.4. Cross-Correlation Analysis

The cross-correlation matrix analysis between the fluctuations of the protein Cα atoms was performed to highlight the coupling of the motions between the protein residues. The cross-correlation (*C_ij_*) coefficients were calculated using the following equation [32,33,34]:

$$C(i,j)=\frac{c(i,j)}{c{\left(i,i\right)}^{1/2}c{\left(j,j\right)}^{1/2}}$$

The positive *C_ij_* values suggest the two residues *i* and *j* moving in the same direction, while the negative *C_ij_* values indicate anti-correlated motions between the two residues *i* and *j*.

### 2.5. MM/GBSA Binding Free Energy Calculations

The molecular mechanics and generalized Born surface area (MM/GBSA) binding free energy calculations were performed to calcuate the binding energy between DAPK1 and CaM [35,36,37]. In general, the binding free energy (Δ*G*_binding_) was calculated by using Equation (1):

(1) $$G_{\mathrm{binding}}=\Delta G_{\mathrm{complex}}-\Delta G_{\mathrm{DAPK}1}-\;\Delta G_{\mathrm{CaM}}$$

where Δ*G*_complex_, Δ*G*_DAPK1_, and Δ*G*_CaM_ are the free energies of the complex, DAPK1, and CaM, respectively. Equation (1) was further calculated as a sum of the gas phase energy (Δ*E*_gas_), the solvation free energy (Δ*G*_solvation_), and the entropy (*T*Δ*S*) based on Equation (2):

(2) $$\Delta G_{\mathrm{binding}}=\Delta E_{\mathrm{gas}}+\Delta G_{\mathrm{solvation}}-T\Delta S$$

The *T*Δ*S* energy item was not calculated due to the extremely long duration of normal mode analysis for large systems [35]. The Δ*E*_gas_ term consists of the van der Waals energy (Δ*E*_vdW_), the electrostatic energy (Δ*E*_ele_), and the internal energy (Δ*E*_int_) based on Equation (3):

(3) $$\Delta E_{\mathrm{gas}}=\Delta E_{\mathrm{vdw}}+\Delta E_{\mathrm{ele}}+\Delta E_{\mathrm{int}}$$

The Δ*G*_solvation_ was calculated using the continuum solvent method, which contains the polar free energy (Δ*G*_GB_) and the nonpolar free energy (Δ*G*_nonpolar_) based on Equation (4):

(4) $${\Delta G}_{\mathrm{solvation}}=\;\Delta G_{\mathrm{GB}}+\;\Delta G_{\mathrm{nonpolar}}$$

The Δ*G*_nonpolar_ was computed using the function of the solvent accessible surface area (SASA) based on Equation (5):

(5) $$\Delta G_{\mathrm{nonpolar}}=\gamma SASA+b$$

where the surface tension parameter *γ* was set to 0.0072 kcal mol^−1^ Å^−2^ and *b* was 0 kcal/mol.

## 3. Results and Discussion

### 3.1. Overview of the Structural Complex of DAPK1–CaM Interactions

In the X-ray crystal structure of the DAPK1–CaM complex (PDB ID: 2X0G) (Figure 1A), the structure of DAPK1 contains the catalytic domain (CD) (residues 3–277) and ARD (residues 278–320) [11]. The ARD contains a N-terminal portion (residues 278–302) that comprises an additional helix (residues 280–288) and a short loop (residues 289–292) in an extended arrangement, and a C-terminal portion (residues 290–320) that adopts a long seven-turn helix. The structure of CaM consists of a N-terminal lobe, referred to as CaM(N) (residues 6–71) and a C-terminal lobe, called as CaM(C) (residues 83–140). A flexible linker connects the CaM(N) to CaM(C). Both the CaM(N) and CaM(C) have four α-helices and two Ca^2+^ ions. In the DAPK1–CaM protein–protein interactions (PPIs) (Figure 1B), the phosphate-binding loop (P-loop) (residues 20–26) and the basic loop (B-loop) (residues 45–55) interact with the CaM(N), the helix αD contacts with the CaM(C), and the C-terminal ARD projects into the well-defined pockets of both the CaM(C) and CaM(N). Obviously, the side chain of Trp305 at the ARD forms a hydrogen bond with the backbone of M124 and is involved in the hydrophobic network formed by Phe92, Ile100, Leu105, Met124, and Phe141 from the CaM(C) (Figure 1C). Despite the important role of Trp305 in the CaM recognition, how the W305Y and W305D mutations differentially affect the DAPK1–CaM complex remains unknown. Next, we performed multiple, μs-length MD simulations to address this important unresolved issue.

### 3.2. System Stability

We examined three systems, including the WT, W305Y, and W305D mutants, to explore the effect of distinct Trp305 mutations on the conformational dynamics of the DAPK1–CaM complex. For each system, MD simulations were conducted in explicit water environments, obtaining multiple μs-length trajectories (i.e., 3 replicates of 1 μs each) and yielding an overall sampling of 9 μs. We simulated multiple and independent μs-length trajectories in order to obtain reliable statistical results.

**Figure 2:**
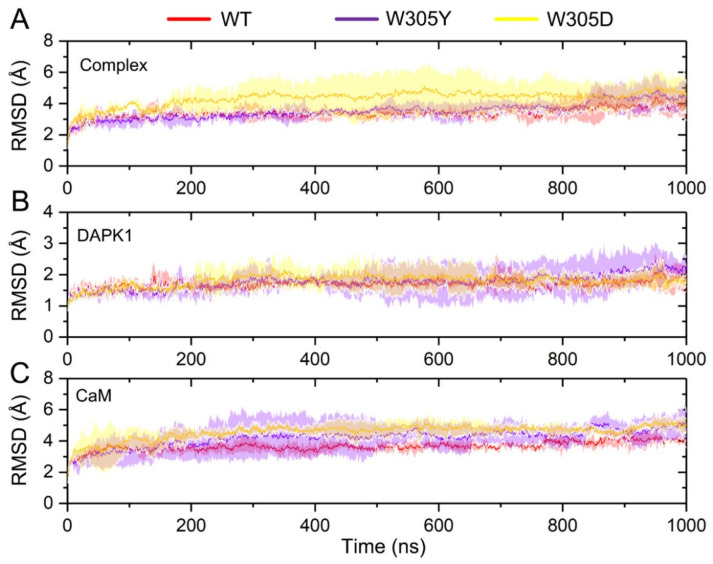
The root-mean-square deviation (RMSD) of the protein Cα atoms as a function of simulation time averaged over three independent replicates for the DAPK1–CaM complex (**A**), the individual DAPK1 (**B**) and CaM (**C**) in the wild-type (WT) (red), W305Y (violet), and W305D (yellow) systems. Transparent shading represents standard deviations.

We analyzed the simulated snapshots at different time points and compared them with the initial crystal structure for each system. The root-mean-square deviation (RMSD) of the protein Cα atoms for the complex, DAPK1, and CaM was monitored through the MD trajectories, which was averaged along the 3 replicates for each system. As shown in Figure 2A, analysis of the RMSD of the protein Cα atoms for the complex showed that each system reached equilibrium after ~200 ns simulation and the WT and W305Y systems achieved a similar stability with a RMSD value of 1.85 ± 0.53 and 1.88 ± 0.37 Å, respectively, while the W305D system experienced a significant conformational plasticity with a RMSD value of 3.73 ± 0.38 Å. To further reveal the origin of the conformational dynamics of the DAPK1–CaM complex, we assessed the RMSD for the individual DAPK1 and CaM. As shown in Figure 2B, analysis of the RMSD of the protein Cα atoms for the DAPK1 revealed that the DAPK1 in each system had a similar stable behavior (i.e., the RMSD reached ~1.5 Å). This result indicated that the Trp305 mutations had a minor effect on the global conformational dynamics of the DAPK1. However, the CaM underwent distinct conformational changes in the different simulated systems. As shown in Figure 2C, after ~200 ns simulations, the RMSD values for the CaM Cα atoms were 3.72 ± 0.38, 4.25 ± 0.69, and 4.85 ± 0.39 Å for the WT, W305Y, and W305D systems, respectively. These data suggested that the CaM was the determinant in the conformational dynamics of the DAPK1–CaM complex and it had the largest flexibility in the W305D system.

### 3.3. W305D Mutation Increases the Inter- and Intra-Domain Correlation Motions

To reveal the interdependent conformational dynamics of the DAPK1–CaM complex among spatially distinct protein domains in the different systems, dynamical cross-correlation analysis was performed [38,39], which was averaged over 3 replicates of MD trajectories for each system. The cross-correlation (*C_ij_*) coefficients ranged from −1 to 1, reflecting the collinear correlation between the two Cα atoms (*i* and *j*). The positive coefficients (*C_ij_* > 0) indicate the movement of the two residues in the same direction and the negative coefficients (*C_ij_* < 0) suggest the movement of the two residues in the opposite direction. The coefficients (*C_ij_* = 0) of zero mean no correlation motions between the two residues. The absolute values of *C_ij_* are proportional to the intensity of the correlated motions of the two residues.

**Figure 3:**
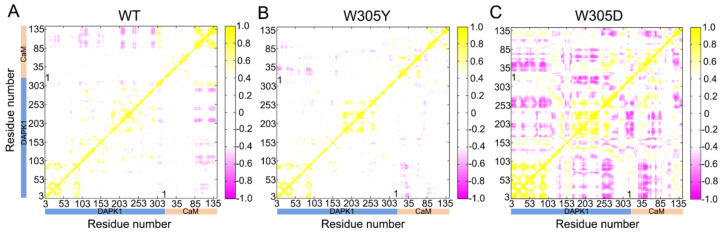
The dynamical cross-correlation matrix (DCCM) plot of the WT (**A**), W305Y (**B**), and W305D (**C**) systems. Anti-correlated/correlated motions with absolute values <0.4 are ignored for clarity and shown in white.

In the WT (Figure 3A) and W305Y (Figure 3B) systems, the *C_ij_* matrix (i.e., a two-by-two plot of the Cα *C_ij_* coefficients) of the DAPK1–CaM complex reveals a similar pattern of correlated/anti-correlated motions of the intra-domain motions of DAPK1 and CaM as well as the inter-domain motions between DAPK1 and CaM. However, in the W305D system (Figure 3C), both the intra-domain motions of DAPK1 and CaM, and the inter-domain motions between DAPK1 and CaM were markedly enhanced compared to those in the WT and W305Y systems. For the intra-domain motions of the individual protein, the W305D mutation increased the anti-correlated motions between the N- and C-lobes of the DAPK1 and between the CaM(N) and CaM(C). In addition to increasing the anti-correlated motions of the intra-domains, the W305D mutation also enhanced the anti-correlated motions of the inter-domains between the DAPK1 and CaM. These results suggest that the W305D mutation yielded an opposed motion between the DAPK1 and CaM, which would cause the disaggregation of CaM with the W305D DAPK1 mutant. However, the W305Y mutant had a similar inter-domain motion between the DAPK1 and CaM with the WT system, implying that CaM can interact with the W305Y DAPK1 mutant.

### 3.4. W305D Mutation Increases the Dynamics of DAPK1–CaM Complex

The dynamical cross-correlation analysis suggested that the DAPK1–CaM complex was more dynamic in the W305D mutant system. To test this hypothesis, principal component analysis (PCA) was carried out [40,41,42]; this can uncover the large-scale collective motions and the conformational interconversion of the DAPK1–CaM complex in the different systems. The PCA results can uncover the directionality and amplitude of protein motions, where the first several principal components (PCs) correspond to large conformational arrangements. PCA analysis has been widely used to decipher the large-amplitude motions of biomacromolecules [43,44,45]. The first two principal components (PC1 and PC2) characterize the conformational space sampled by the DAPK1–CaM complex during MD simulations. Importantly, in order to ensure the consistency of the motions of the principal components in the same collective coordinate space and to identify the differences in the essential dynamics of the DAPK1–CaM complex, the simulated snapshots were overlapped with the same reference structure (i.e., the initial crystal structural complex).

**Figure 4:**
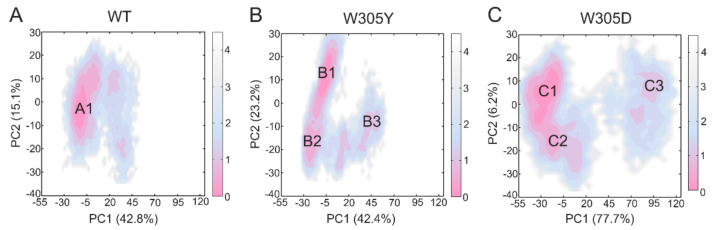
The free energy landscape of the first and second principal components (PC1 vs. PC2) from MD simulations of the WT (**A**), W305Y (**B**), and W305D (**C**) systems. The unit of free-energy values is kcal/mol. The representative DAPK1–CaM structural complex for the WT (A1), W305Y (B1, B2, and B3), and W305D (C1, C2, and C3) are shown.

The PCA results for the DAPK1–CaM complex showed that the combined PC1 and PC2 occupied ~70% of the variance for both the WT (Figure 4A) and W305Y (Figure 4B) systems and ~80% of the variance for the W305D system (Figure 4C). Moreover, the free energy landscapes of the PC1 and PC2 were plotted onto a two-dimensional space to show the conformational space sampled by the different DAPK1–CaM complexes. In both the WT and W305Y systems, the PC1 vs. PC2 plots suggested a similar distribution of the conformations. For instance, the PC1 and PC2 values in the WT system ranged from ~−30 to ~45, and from ~−30 to ~20, respectively. In the W305Y system, they ranged from ~−30 to ~55, and from ~−30 to ~30, respectively. However, in the W305D system, the PC1 vs. PC2 plots revealed a remarkable increase in the amplitude of motions compared to both the WT and W305Y systems. For example, the PC1 and PC2 values in the W305D system ranged from ~−55 to ~120, and from ~−40 to ~20, respectively. Taken together, these data indicated that the W305D system sampled a broad distribution of conformations along the MD simulations, highlighting the enhanced conformational dynamics of the DAPK1–CaM complex induced by the W305D mutation.

### 3.5. W305D Mutation Disturbs the DAPK1–CaM Interface

The two-dimensional energy landscapes of the PC1 and PC2 revealed that the WT, W305Y, and W305D systems had one (A), three (B1, B2, and B3), and three (C1, C2, and C3) conformational sub-basins (Figure 4), respectively. We next extracted the structures from these sub-basins and conducted clustering analysis on these structures for each system [46,47]. Then, the representative structure extracted from each sub-basin from the W305Y and W305D mutant systems was aligned with that from the WT system to unravel the detailed conformational changes in the protein domains triggered by the Trp305 mutations.

**Figure 5:**
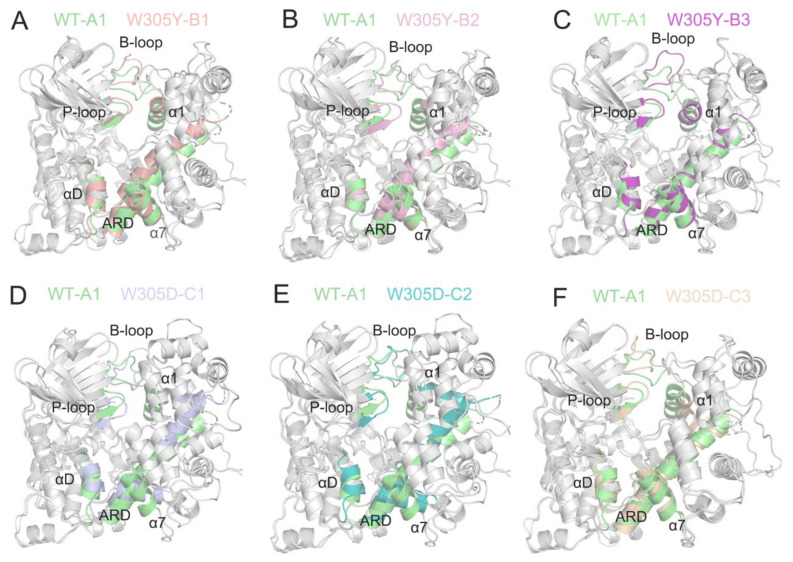
(**A**–**C**) The superimposition of the three representative structures (B1, B2, and B3) from the W305Y mutant system on the representative structure A1 from the WT system. (**D**–**F**). The superimposition of the three representative structures (C1, C2, and C3) from the W305D mutant system on the representative structure A1 from the WT system.

As shown in Figure 5A, the superimposition of the three representative structures (B1, B2, and B3) from the W305Y mutant system to the representative structure A1 from the WT system show that the interface of the DAPK1–CaM complex in the W305Y mutant, including the B-loop, P-loop, helix αD, and the N-terminal ARD from the DAPK1 and the helices α1 and α7 from the CaM, underwent little conformational changes compared to those in the WT system. However, the interface of the DAPK1–CaM complex in the W305D mutant exhibited noticeable conformational changes compared to that in the WT system (Figure 5B). As shown in Figure 4C, the representative structures (C1 and C2) occupied the vast majority of conformations of the DAPK1–CaM complex for the W305D system. The most remarkable differences found in the CaM were the conformational arrangements of the helices α1 and α7, while the DAPK1 showed subtle conformational differences between the C1/C2 from the W305D system and A1 from the WT. Thus, the resulting departure of the helices α1 and α7 of the CaM from the interface of the DAPK1–CaM complex in the W305D mutant would impair the interactions with the B-loop, P-loop and helix αD from the DAPK1. For the representative structure C3, structural overlapping of the C3 from the W305D system with the A1 from the WT system exhibited that the interface of the DAPK1–CaM complex in the W305D system was similar to that in the WT (Figure 5B). However, the representative structure C3 occupied a minor population of the conformational ensemble of the W305D DAPK1–CaM complex (Figure 4C), implying that this structure only existed at the beginning of the MD simulations. Collectively, analyses of conformational clusters in the different systems suggested that the W305D mutation disrupted the interface of the DAPK1–CaM complex, while the W305Y mutation had a minor effect on the interface.

### 3.6. W305D Mutation Impairs the DAPK1–CaM Interactions

To further quantitatively assess the energetics of the DAPK1–CaM interactions in the three different systems, the molecular mechanics and generalized Born surface area (MM/GBSA) binding free energy calculations were performed [48,49,50,51,52,53,54]. As shown in Table 1, the binding free energies for the WT, W305Y, and W305D systems were −116.44 ± 15.60, −118.77 ± 16.86, and −92.38 ± 17.54 kcal/mol, respectively. The lower binding free energy implied a stronger interaction between DAPK1 and CaM. A higher binding free energy for the W305D mutant showed that the CaM binding was not as favored as it was in the WT and W305Y systems, while the WT and W305Y systems had a similar binding free energy. This result was consistent with the in vitro experiments [11]. A detailed analysis of the individual energy contributions revealed that the electrostatic energy (Δ*E*ele) was largely responsible for the increased binding free energy of CaM to the W305D DAPK1, since the difference between the Δ*E*ele before and after the mutation increased most significantly.

**Figure 6:**
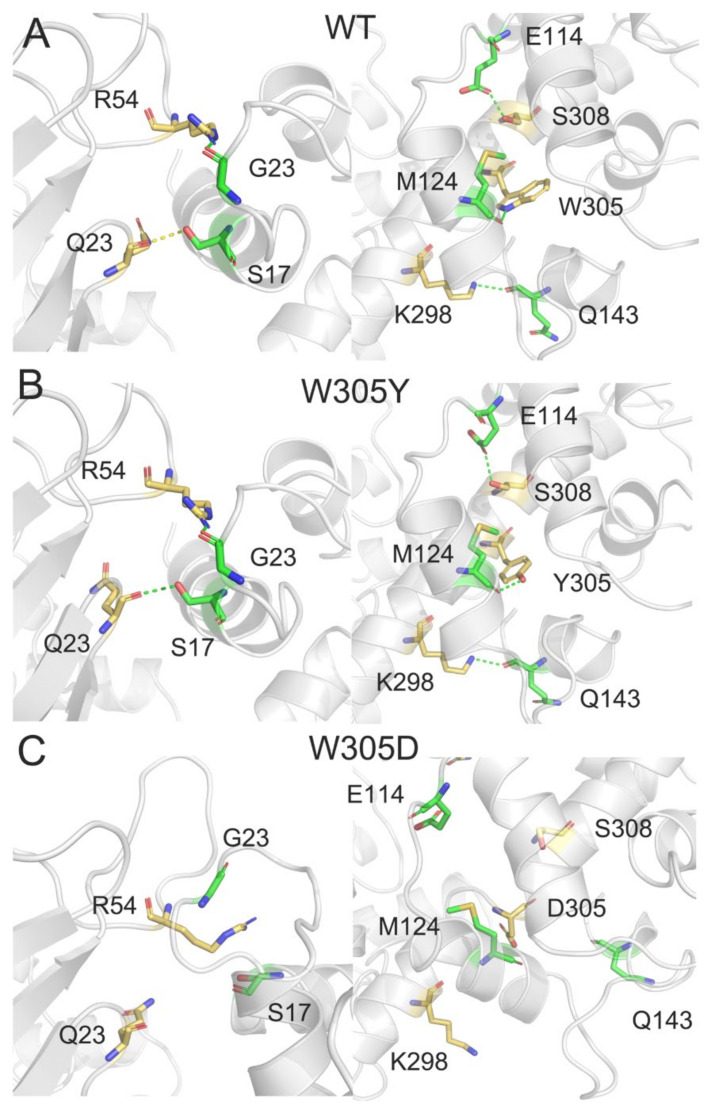
The hydrogen bonds between the interface of DAPK1 and CaM in the WT (**A**), W305Y (**B**), and W305D (**C**) systems. Hydrogen bonds are shown by green dotted lines.

In order to unravel the molecular mechanism of the decreased binding free energy in the W305D mutant, we used PISA (Proteins, Interfaces, Structures and Assemblies) [55] to evaluate the DAPK1–CaM interactions in the WT, W305Y, and W305D systems. The hydrogen bonds and salt bridges were analyzed in the three different systems, using the most populated structural complex from the conformational ensemble for each system as an input coordinate (A1 for the WT, B1 for the W305Y system, and C1 for the W305D system). Because the W305D mutation largely reduced the electrostatic energy between DAPK1 and CaM, we focused on the hydrogen bonding and salt bridge interactions between the interface of DAPK1 and CaM. In both the WT (Figure 6A) and W305Y (Figure 6B) systems, five hydrogen bonds were formed between the DAPK1 and CaM. For example, the backbone of Gln23 at the P-loop of DAPK1 forms a hydrogen bond with the side chain of Ser17 at the helix α1 of CaM; the side chain of Arg54 at the B-loop of DAPK1 forms a hydrogen bond with the backbone of Gly23 at the helix α1 of CaM; the side chains of Lys298 and Ser308 at the C-terminal ARD of DAPK1 are involved in hydrogen bonds with the backbone of Gln143 at the helix α8 and the side chain of Glu114 of CaM, respectively. In the WT, the side chain of Trp305 at the C-terminal ARD forms a hydrogen bond with the backbone of Met124 at the helix α7 of CaM, while in the W305Y mutant, the side chain of the mutated Tyr305 engages in a hydrogen bond with the backbone of Met124. Overall, the hydrogen bonding interactions between the interface of DAPK1 and CaM in the WT system are conserved in the W305Y mutant system. Noticeably, in the W305D mutant system (Figure 6C), all the above hydrogen bonding interactions were disrupted.

**Figure 7:**
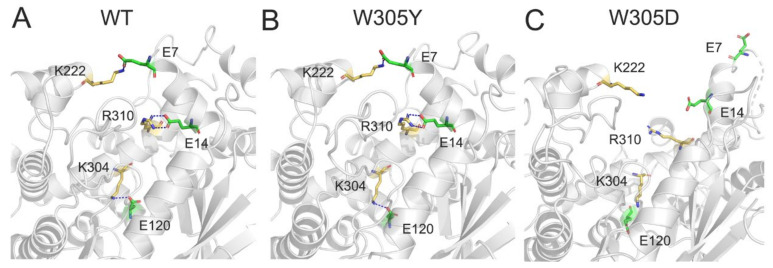
The salt bridges between the interface of DAPK1 and CaM in the WT (**A**), W305Y (**B**), and W305D (**C**) systems. Salt bridges are shown by blue dotted lines.

For the salt bridge interactions, in both the WT (Figure 7A) and W305Y (Figure 7B) systems, four salt bridges were formed between the DAPK1 and CaM. For example, one salt bridge was from the side chain of Lys222 of DAPK1 and the side chain of Glu7 at the helix α1 of CaM, two salt bridge were from the side chain of Arg310 at the C-terminal ARD of DAPK1 and the side chain of Glu14 at the helix α1 of CaM, and one salt bridge was from the side chain of Lys304 at the C-terminal ARD of DAPK1 and the side chain of Glu120 at the helix α7 of CaM. However, in the W305D mutant system (Figure 7C), the four abovementioned salt bridges were lost. Collectively, the loss of the hydrogen bonding and salt bridge interactions between the interface of DAPK1 and CaM in the W305D mutant would attenuate the DAPK1–CaM interactions. These data were in agreement with the in vitro biochemical experiments [11], where the W305Y mutation only moderately decreased CaM binding, but the W305D mutation significantly reduced CaM binding. As a result, the DAPK1 W305D mutant exhibited an obvious increase in Ser308 autophosphorylation because of the exposure of Ser308 to the DAPK1’s upstream PI3K kinase, which can phosphorylate Ser308.

## 4. Conclusions

In the present study, we investigated the molecular mechanism underlying the key role of Trp305 in modulating DAPK1 activity using multiple μs-length MD simulations, dynamical cross-correlation analysis, principal component analysis, and MM/GBSA binding free energy calculations. The overall conformational dynamics of the DAPK1–CaM complex were not obviously affected by the W305Y mutation, but were significantly influenced by the W305D mutation. The dynamical cross-correlation analysis revealed that the W305D mutation markedly enhanced the anti-correlated motions between the DAPK1 and CaM compared to both the WT and W305Y systems, which would disengage the CaM from the W305D DAPK1 mutant. The PCA results revealed that the W305D mutant sampled a broader distribution of conformational space relative to both the WT and W305Y systems. Moreover, the MM/GBSA binding free energy calculations showed that the W305D mutation attenuated the binding of CaM to the DAPK1 and the loss of CaM binding to the W305D DAPK1 mutant mainly derived from the electrostatic energy (Δ*E*ele). Detailed structural analyses further revealed that the five hydrogen bonds and the four salt bridges between the interface of DAPK1 and CaM in the WT and W305Y systems were disrupted in the W305D system. These hydrogen bonds and salt bridges include Q23^DAPK1^–S17^CaM^, R54^DAPK1^–G23^CaM^, K298^DAPK1^–Q143^CaM^, W305D/Y^DAPK1^–M124^CaM^, S308^DAPK1^–E114^CaM^, K222^DAPK1^–E7^CaM^, K304^DAPK1^–E120^CaM^, and R310^DAPK1^–E14^CaM^. Overall, the W305D mutation disrupted the interface of the DAPK1–CaM complex largely lined by the P-loop, the B-loop, and the C-terminal ARD from the DAPK1 and the helices α1 and α7 from the CaM. The resultingly weakened interactions between the W305D DAPK1 and CaM deduced from MM/GBSA calculations would enhance the exposure of the Ser308, which would be more accessible by the DAPK1’s upstream PI3K kinase to phosphorylate Ser308. The obtained results may be helpful in providing insights into the key role of Trp305 in the regulation of CaM-mediated modulation of DAPK1 activity.

## Figures and Tables

**Table 1:** Binding free energy (kcal/mol) between the different DAPK1 states and CaM.

	WT	W305Y	W305D
Δ*E*_vdW_	−189.72 ± 11.76	−184.08 ± 11.64	−189.00 ± 10.64
Δ*E*_ele_	−1743.98 ± 106.00	−1732.59 ± 120.61	−1357.90 ± 118.32
Δ*G*_SA_	−29.49 ± 1.36	−30.30 ± 1.60	−29.34 ± 1.38
Δ*G*_GB_	1817.26 ± 98.24	1797.89 ± 112.31	1454.53 ± 108.30
Δ*G*_binding_	−116.44 ± 15.60	−118.77 ± 16.86	−92.38 ± 17.54

## Data Availability

Not applicable.
